# Effective utilization of attentional resources in postural control in athletes of skill-oriented sports: an event-related potential study

**DOI:** 10.3389/fnhum.2023.1219022

**Published:** 2023-08-24

**Authors:** Jiacheng Chen, Alex Pak Ki Kwok, Yanan Li

**Affiliations:** ^1^College of Education for the Future, Beijing Normal University, Zhuhai, China; ^2^Data Science and Policy Studies Programme, Faculty of Social Science, The Chinese University of Hong Kong, Hong Kong, Hong Kong SAR, China; ^3^Physical Education Department, Zhuhai Campus of Jinan University, Zhuhai, China

**Keywords:** skill-oriented sports, postural stability, dual-task, event-related potentials, attentional resources

## Abstract

**Objective:**

Postural control plays a key role in skill-oriented sports. Athletes of skill-oriented sports (hereinafter referred to as “skilled athletes”) usually showed better control ability compared with non-athletes. However, research focused on the single postural task, rarely considering the actual situation in skill-oriented sports in which other processes, such as cognitive control, frequently accompany postural control. This study aims to explore how skilled athletes control their posture under the dual-task situation and use limited attentional resources.

**Method:**

A total of 26 skilled athletes and 26 non-athletes were required to perform the postural control and N-back tasks simultaneously. Center of pressure (COP) trajectory, reaction times (RTs), and discriminability (d′) of N-back tasks were recorded and evaluated, along with event-related potentials, including N1 (Oz, PO7, and PO8), P2 (Fz, FCz, Cz, and Pz) components, and the spectral power of alpha band.

**Results:**

Skilled athletes demonstrated more postural control stability and a higher d′ than non-athletes in all dual tasks. Besides, they showed enhanced N1, P2 amplitudes and reduced alpha band power during dual-tasking. Notably, in skilled athletes, a significant negative correlation between N1 amplitude and d′ was observed, while significant positive correlations between alpha band power and postural control performance were also identified.

**Conclusion:**

This study investigates the potential advantages of skilled athletes in postural control from the view of neuroscience. Compared to non-athletes, skilled athletes could decrease the consumption of attentional resources in postural control and recruit more attentional resources in stimulus discrimination and evaluation in cognitive tasks. Since the allocation of attentional resources plays a crucial part in postural control in skilled athletes, optimizing the postural control training program and the selection of skilled athletes from a dual-task perspective is important.

## Introduction

Postural control is the regulation of the body’s position in space to maintain balance and orientation (Shumway-Cook and Woollacott, 2000; Woollacott and Shumway-Cook, 2002), which plays a vital role in good performance in skill-oriented sports. Skill-oriented sports belong to closed-skill sports, which require high body control and reflect the action’s difficulty, beauty, and elegance, e.g., gymnastics, rhythmic gymnastics, and martial arts routin. Previous studies also have demonstrated a positive relationship between postural control ability and sports performance (Gautier et al., 2008; Costa et al., 2017; Marcolin et al., 2018).

Indeed, postural control is conscious processing that requires attentional resources. Concurrent cognitive processes would compete for limited attentional resources (Vuillerme and Nougier, 2004). Studies found a decline in concurrent spatial memory task performance while maintaining a steady posture (Kerr et al., 1985; Chen et al., 2018). If postural control is deprived of sufficient resources, stability will be weakened, meaning that the people are at risk of falling (Tombu and Jolicoeur, 2003; Ruffieux et al., 2015). However, postural control occurs in conjunction with the regulation of cognitive processes (Woollacott and Shumway-Cook, 2002; Fraizer and Mitra, 2008). For example, when performing a set of movements, rhythmic gymnasts should remember and update the following movements and cohesions while focusing on the quality of motor control to achieve outstanding performance. They should also inhibit ongoing improper actions while maintaining their current posture. Currently, most research focuses on athletes’ postural control in a single task, rarely considering the real scenario in skill-oriented sports where postural control is frequently accompanied by other tasks such as update processing or inhibitory control (Asseman et al., 2008; Isableu et al., 2017). Therefore, it is vital to consider postural control under the influence of cognition to evaluate the characteristics of postural control abilities in athletes of skill-oriented sports (hereinafter referred to as “skilled athletes”). This would help to optimize the postural control training program and the selection of skilled athletes.

Dual-tasking is commonly employed to study the effects of cognition during balance performance, in which participants undertake a secondary task while completing the postural control task. Vuillerme and Nougier (2004) found that the reaction time (RT) for the unpredictable auditory stimulus grew longer during dual-tasking as the postural task’s difficulty increased. Besides, this effect was smaller for gymnasts than non-gymnasts, suggesting a reduced dependency on attentional demand of postural control during dual-tasking in gymnasts (Vuillerme and Nougier, 2004). However, little research has further investigated this issue. More and more sports professionals have become interested in brain imaging to further understand the neural mechanisms underlying sporting performance, including its acquisition and execution (Park et al., 2015). The plastic adaptive changes in the neuronal circuits of the brains of athletes have been widely investigated in studies, which shows that daily physical training leads to changes in the neuroplasticity of cortical regions, such as the sensory and motor cortex (MI) (Hanggi et al., 2010; Naito and Hirose, 2014), and frontal areas (Kim et al., 2008). These findings suggest that there is a reduction in neural activity in certain brain regions called “neural efficiency” as a particular skill becomes more automated and less controlled (Debarnot et al., 2014), i.e. In other words, they achieved the best results with minimal energy consumption (Nakata et al., 2010). Thus, we believe the differential and specialized neural processing would be found in skilled athletes during the postural control dual-task.

Event-related potential (ERP) is one of the oldest methods for assessing the relationship between the brain and behavior and provides a direct real-time measure of neural activity. In the dual-task paradigm, N1 and P2 (Fz, FCz, Cz, Pz, Oz) are often used to reflect the allocation and utilization of attentional resources. Sibley et al. (2010) examined how the brain responds when perturbation is applied to postural control. The perturbation-evoked cortical potentials N1 component was significantly greater at high ground level than at low level. In contrast, Maeno et al. (2004) revealed that the parietal N1 and frontoparietal P2 were greater in two-foot standing than in single-foot standing. Cheng-Ya et al. (2016) also required participants to perform a force-matching task while maintaining balance at a target angle. Their results showed increased balancing demand was associated with greater frontal cluster coefficients. Besides, there was an anterior shift of processing resources toward frontal executive function.

Spectral power in different EEG frequency bands like alpha power (8–12 Hz) is sensitive to arousal, resource allocation or workload (Fink et al., 2005). Studies revealed that alpha band activity decreased with increased task complexity and mental workload (Gevins and Smith, 2003; Parasuraman and Wilson, 2008; Brouwer et al., 2012). Besides, research also showed the possibility of using alpha band power to evaluate the performances of professional athletes (Babiloni et al., 2008). All these studies suggested that a greater balancing load resulted in a greater allocation of attentional resources and that the frontal cortex is the important region for postural control dual-task processing. Still, most studies focused only on non-athletes and ignored the athletes of skill-oriented sports with better postural control abilities. Therefore, this study specifically wanted to explore how skilled athletes with better postural control distribute and use their limited attentional resources during dual-tasking.

The cross-domain competition model suggested that concurrent secondary tasks would increase the demand for attentional resources (Tombu and Jolicoeur, 2003; Ruffieux et al., 2015). As a part of executive function, working memory is crucial for cognitive processing related to the prefrontal cortex and occupies some attentional resources (Morris and Jones, 2011). N-back task is a commonly employed technique to assess working memory, involving the storage, maintenance, and manipulation of information, as well as inhibitory control (Oberauer, 2005; Jaeggi et al., 2015; Shalchy et al., 2020). ERP offers excellent temporal resolution, making it well-suited for studying the temporal dynamics of working memory processes and the allocation of attentional resources during the N-back task. The N1 component of ERP is related to the orientation of attention to a task-relevant stimulus (Luck et al., 1990; Herrmann and Knight, 2001). Increased N1 amplitudes observed in the N-back task can indicate more efficient attentional orientation during the early processing stage. Increased N1 amplitudes observed in the N-back task can indicate more efficient attentional orientation during the early processing stage. Additionally, the P2 component is linked to stimulus detection and attentional processes (Risto, 1992), particularly in the initial allocation of attention during the N-back task (Lenartowicz et al., 2010). Therefore, the N-back task is considered appropriate for investigating working memory processes using ERP recordings. It provides valuable insights into the competition for attentional resources between working memory and postural control. Hence, this study took the N-back task as a secondary task in the dual-task paradigm.

In detail, the present study aimed to investigate the effect of working memory on balance control in skilled athletes vs. non-athletes. It was hypothesized that skilled athletes would demonstrate greater control stability compared with non-athletes. Besides, in their ERP components, we expected that the typical attention-related components N1 and P2 would be greater in skilled athlete than non-athletes. Additionally, based on previous studies that found correlations between the alpha band and postural control (Neuper and Pfurtscheller, 2001), this study hypothesized that a lower alpha band power would be found among skilled athletes during dual-task compared with non-athletes.

## Materials and methods

### Participants

A three-way repeated measures design was employed, with groups (skilled athletes vs. non-athletes) serving as the between-subjects factor, the postural control task (FT, TD, SL), and cognitive task (1-back, 3-back) serving as the within-subject factors. The G*power software was utilized to estimate the required sample size for the study. The specific parameters set in G*power were as follows:

• Effect size: The effect size was set to 0.4, based on prior research in a similar domain. However, it should be acknowledged that additional information about the rationale behind this effect size estimation would have been beneficial (Chen et al., 2019).
• Significance level: A significance level of 0.05, commonly used in scientific research, was chosen.
• Statistical test power: The aim was to achieve a statistical test power of 0.95, indicating a desire for a high probability of detecting a true effect if it exists.

Based on these parameters, G*power initially indicated that a sample size of at least 12 participants would be sufficient. However, considering the complexity of the behavioral and ERP tasks, more participants were recruited than the minimum requirement.

For the skilled athlete group, 26 athletes from gymnastics, martial arts routin, and sports acrobatics, were recruited from the Shanghai University of Sport professional team, with a mean age of 20.08 years (SD = 0.25) for 2 men, and 19.88 years (SD = 0.71) for 24 women. The inclusion criteria included (1) having more than 5 years of professional training (> 12 h/week) before joining the college team, (2) maintaining skill training (> 5 h/week) during college, (3) these athletes are above the second level of the national standard, (4) having no injuries or lesions of the knee, hip or ankle recently.

For the non-athlete group, 26 college students were recruited from the Shanghai University of Sport, with a mean age of 20.21 years (SD = 0.38) for 2 men, and 20.56 years (SD = 0.64) for 24 women. The inclusion criteria included (1) no sports training experience, (2) having watched the gymnastics, martial arts routin, sports acrobatics matches less than five times during the past 5 years, (3) having no regular exercise routines. Table 1 shows characteristics regarding the age, training experience, and anthropometric information of the two groups.

**TABLE 1:** The age, training experience and somatic information of two groups.

	Skilled athletes					Nonathletes				
	N	Mean	Minimum	Maximum	SD	N	Mean	Minimum	Maximunm	SD
Age (years)	26	20.2	19.4	20.6	0.5	26	19.7	19.3	20.5	0.9
Height (cm)	26	166.1	163.6	168.1	2.1	26	165.2	163.3	167.2	2
Body mass (kg)	26	52.2	49.8	54	2.3	26	51.6	48.5	53.5	2.1
Training experience (years)	26	8.7	7	9.4	1.1	26				

Regarding the male-to-female ratio in the sample, it is important to note that while female athletes make up the majority in skill-oriented sports, male participants were included to achieve a representative sample reflecting the population of athletes in these sports. Despite the smaller number of male participants, their inclusion allows for a comprehensive understanding of postural control in skill-oriented sports and captures the representation of both genders. Outlier tests conducted on all participants, including the two males, confirmed that their data fell within the normal range. Therefore, the data of male participants were deemed appropriate for analysis.

Prior to participation, all subjects provided written informed consent. They were also required to complete a survey concerning their demographic information. After the experiment, they were reimbursed RMB¥150 for their participation. All subjects reported not smoking, having a normal or corrected-to-normal vision, being right-handed, and being free of mental and physical illnesses.

### Task procedures

The participants were given instructions for the dual-task paradigm, which required them to perform the postural control task on a balancing apparatus while simultaneously completing the N-back task.

**FIGURE 1:**
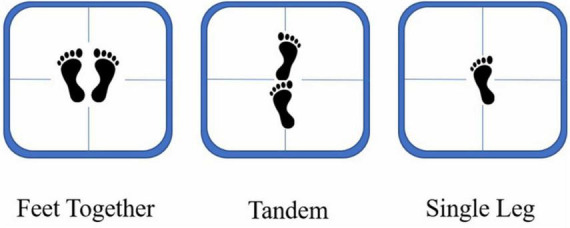
Illustration of the three types of standing conditions: feet together **(left)**, tandem **(center)**, and single leg **(right)**.

The main task (of the dual-task) was the postural control task. There were three challenging standing conditions in this task: feet together (FT), tandem (TD), and single leg (SL) (Figure 1). For the FT, participants were instructed to stand on a balancing apparatus with their eyes open and their hands hanging freely at their sides with their feet one shoulder width apart. For the TD condition, participants were required to stand with their feet in tandem on the center line of the balancing apparatus, with the toe of the left foot near the heel of the right foot. In contrast, for the SL condition, participants were asked to stand by their dominant leg (reported in the questionnaire) in the center of the balancing apparatus, with their other leg bent at the knees and the instep positioned in the middle of the gastrocnemius of the dominant leg. During the experiment, participants were required to maintain a straight body position, relax with their eyes open, try their best to maintain balance, and minimize swaying.

**FIGURE 2:**
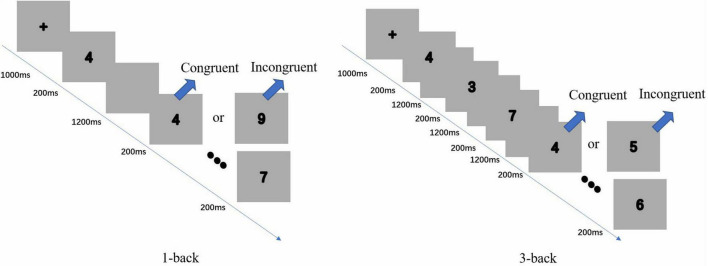
Illustration of the two difficulties of the N-back task: 1-back **(left)** and 3-back **(right)**. Two tasks consist of consecutive numbers, which presented 200 ms in each block, and a blank screen of 1,200 ms appeared between each trial.

The secondary task is N-back. This exercise included two challenges: 1-back and 3-back (see Figure 2). The stimulus was a single element (numbers 1 to 9) that appeared continuously and randomly on the screen. Participants had to compare the current number to the Nth number that appeared beforehand. If the two numbers matched, the participant needed to click the mouse left button with their right index finger. In contrast, if the two numbers did not match, they needed to click the mouse right button with their left index finger. In 1-back, participants started with the second number offered and continuously compared the current number to the previous number shown. In 3-back, they began with the fourth number presented and continuously compared the current number to the third number that appeared ahead. For example, they needed to compare the fifth number to the second and the sixth number to the third number.

In N-back, each block began with a 1,000-ms presentation of a cross on the screen, a 200-ms presentation of a number, and a 1,200 ms presentation of a blank screen until the next number appeared. All participants had an opportunity to practice each task 15 times. These practice data were excluded from the analysis. The formal experiment had four blocks of 120 trials for each N-back task, with 40 inconsistent and 80 consistent trials in each block. Moreover, there was a 30-s interval between each block. All tasks were programmed using E-prime 2.0. The stimuli were displayed on a laptop with a 13-inch screen.

In the dual-task paradigm, participants had to perform the postural control task and N-back task at the same time. When the experiment began, they stood on the balancing apparatus with the specified posture. All participants stood 80 cm in front of a screen placed at eye level, and the viewing angle was 0.75 degrees. The participants lowered their hands naturally, each holding a mouse. They were challenged to divide their attention between the two tasks, maintaining balance while performing the N-back quickly and accurately. When the N-back began, the balancing apparatus recorded the COP trajectory synchronously. There were four 50-s blocks in each standing posture, with a 30-ms gap between each block. All participants must complete six dual-task sets: FT-1-back, TD-1-back, SL-1-back, FT-3-back, TD-3-back, and SL-3-back. The order of the six dual-task sets was counterbalanced across participants.

### Data collection

The time series data of the COP trajectory, sampled at a frequency of 100 Hz, was recorded using Super Balance (ACMEWAY, Beijing, China). The behavioral response data were collected using E-prime 2.0. Meanwhile, the electroencephalographic (EEG) data were recorded using a Brain Vision EEG system (Brain Products GmbH, Gilching, Germany) referenced against FCz with a 64-channel amplifier and 500 Hz sampling frequency.

Continuous EEG measurements were taken and averaged from the right and left mastoids, and the ground electrode was placed in the mid-forehead. Both horizontal and vertical eye movement blinks were recorded. Electrode impedances for all electrooculogram and EEG electrodes were kept below 10 kΩ.

### Data and statistical analyses

#### Normality test

A normality test was conducted on all the collected data to ensure its distribution. The Shapiro–Wilk (W–S) test was utilized for this purpose. The obtained W–S significance levels were greater than 0.05, indicating a normal distribution in the data. Outliers were also inspected using boxplots, and no values needing removal were found. Following these procedures, formal statistical analyses were performed on the data.

#### Behavioral data

For the postural control task and group, the envelope area (ENV), whole path length (WPL), mean speed (MS), and path length per unit area (PLUA) of the COP trajectory were calculated for each task and group. For the N-back task, three dependent variables were gathered for calculation. The first two variables were response accuracy (ACC) and response time (RT). They were calculated for 1-back and 3-back. The third one was the discriminability index (d′), denoted as Z_hit_−Z_false alarm_ according to the signal detection theory (Stanislaw and Todorov, 1999). For the subject with a 100% hit rate, this study used the formula: “Hit rate = (hit−5)/trials of consistent condition” to revise the hit rate. The modified hit rate was then transferred to the Z score to calculate the discriminability index (Stanislaw and Todorov, 1999). A three-way repeated measures analysis of variance (ANOVA) was performed for ACC, RT, ENV, WPL, MS, and PLUA, with groups (skilled athletes vs. non-athletes) serving as the between-subjects factor and the postural control task (FT, TD, SL) and cognitive task (1-back, 3-back) serving as the within-subject indexes.

#### ERP/EEG data

The ERP and EEG data were analyzed using the Analyzer software provided by the Brain Vision EEG system. Offline re-referencing of all channels was performed by averaging the two mastoid electrodes. To correct for eye movements and blinks, the independent component analysis (ICA) was utilized, selecting the Semiautomatic option and Meaned Slope Algorithm with an interval length of 50 ms. Trials with amplitudes exceeding ± 100 μV were excluded, resulting in 112 epochs for 1-back and 115 epochs for 3-back analysis. A digital bandpass filter with a frequency range of 0.1 Hz to 30.0 Hz was applied to the recorded EEG data to reduce low-frequency content unrelated to the components of interest. Additionally, a notch filter at 50 Hz was implemented to remove powerline noise. Each EEG data epoch was divided into 1,000-ms segments, covering the period from 200 ms before the stimulus onset to 800 ms after the stimulus onset. To account for steady brain activity, the peak amplitude of each ERP component was determined by averaging ERP data for each group and condition. Only trials with correct responses in both tasks were included for further analysis. Attention-related ERP components N1 and P2 were analyzed (Egetemeir, 2006). Three electrode sites (Oz, PO7, and PO8) were chosen to investigate the N1 component. For examination of the P2 component, four midline electrodes (Fz, FCz, Cz, and Pz) were used. According to the grand average waveforms and topographic scalp distributions, this study analyzed three time windows: 130–190 ms for N1 and 130–190 ms for P2. These peak amplitudes were placed into a three-way repeated measures ANOVA with groups (skilled athletes vs. non-athletes) as the between-subject factor, electrode sites (3/4 electrode sites), and N-back (1-back, 3-back) as the within-subject factor.

For EEG data analysis, each block’s initial and final 5 s were omitted, leaving a 40-s time window for further processing. Therefore, the total time window for each task was 160-s. It was then divided into 1-s epochs. The fast Fourier transform (FFT) of Welch method was adopted and Hanning window was added to estimate the power spectrum for each task and electrode site. This study mainly analyzed the alpha (9–12 Hz) power band (Edward et al., 2011). The three areas of interest were included: parietal area (P3, Pz, and P4), parieto-occipital area (PO3, POz, and PO4), and occipital area (O1, Oz, and O2). Finally, the averaged power spectrum of three electrode sites in each region of interest served as the power spectrum for each area. Alpha power was evaluated in three different postural conditions using a three-way repeated measures ANOVA with groups (skilled athletes vs. non-athletes) as the between-subject factor and interest area (parietal, parieto-occipital, and occipital) and N-back (1-back, 3-back) as the within-subject factors. Another three-way repeated measures ANOVA was performed to evaluate the alpha power in two groups under different tasks, with interest area (parietal, parieto-occipital, and occipital), standing posture (FT, TD, and SL) and N-back (1-back, 3-back) as the within-subject factor.

In addition, this study calculated the bivariate correlation between N1 amplitude at the occipital electrodes (Oz, PO7, and PO8) and d′ to evaluate the relationship between cognitive processes and attentional resources. It also evaluated the bivariate correlation between alpha power and COP trajectory to examine the relationship between postural control and brain activity (ENV).

For all analyses (behavioral and ERP/EEG data), *p*-values less than 0.05 were deemed statistically significant. The *p*-values ranging from 0.05 to 0.08 were considered marginally significant. Degrees of freedom were corrected using the Greenhouse–Geisser method. The effect size for each comparison was reported. The partial eta-squared (η_p_^2^) was used to measure the effect size. The least significant difference approach was used for *post-hoc* tests of significant main effects. The test was highly sensitive. It was possible to identify slight differences in the mean value of each level.

## Results

### Behavioral results

#### Postural control

The COP trajectory: ENV, WPL, MS, and PLUA were included in statistical analyses (Table 2).

**TABLE 2:** Postural control data associated with 2 N-back tasks during dual-task.

Measures	Task	Posture	Athletes (*n* = 26)	Median	W-S	Nonathletes (*n* = 26)	Median	W-S	Cohen’s *d*
ENV (mm^2^)	1-Back	FT	67.24 ± 44.36	55.61	0.25	133.72 ± 88.62	110.44	0.79	0.95*
		TD	186.18 ± 119.57	149.1	0.49	515.10 ± 706.45	318.11	0.51	0.65*
		SL	246.24 ± 113.44	209.67	0.48	444.08 ± 230.91	390.23	0.68	1.09**
	3-Back	FT	63.67 ± 64.28	46.05	0.09	136.54 ± 114.37	91.17	0.97	0.79*
		TD	176.68 ± 156.57	119.82	0.34	341.05 ± 202.66	272.76	0.55	0.91*
		SL	321.29 ± 609.70	174.24	0.49	424.03 ± 212.48	425.78	0.45	–
WPL (mm)	1-Back	FT	766.07 ± 104.19	740.87	0.19	839.20 ± 289.50	770.69	0.12	–
		TD	1200.83 ± 218.97	1190.18	0.48	1468.06 ± 392.92	1387.32	0.46	0.84*
		SL	1477.84 ± 273.82	1476.85	0.59	1807.74 ± 537.20	1653.48	0.6	0.77*
	3-Back	FT	799.09 ± 131.77	752.91	0.41	894.26 ± 501.17	793.15	0.45	–
		TD	1180.60 ± 162.46	1183.03	0.43	1416.45 ± 416.35	1301.21	0.23	0.75*
		SL	1467.61 ± 290.01	1384.53	0.48	1764.85 ± 485.39	1708.77	0.15	0.94*
MS (mm/s)	1-Back	FT	12.77 ± 1.70	12.48	0.26	14.15 ± 5.23	12.93	0.19	–
		TD	20.05 ± 3.70	19.87	0.38	24.72 ± 7.14	23.68	0.43	0.82*
		SL	25.05 ± 5.00	24.79	0.95	30.76 ± 9.45	28.15	0.19	0.76*
	3-Back	FT	12.77 ± 1.92	12.63	0.48	15.15 ± 9.00	13.18	0.33	–
		TD	20.01 ± 2.74	19.84	0.31	23.68 ± 7.34	22	0.25	0.66*
		SL	24.73 ± 5.09	23.39	0.33	30.01 ± 8.67	28.8	0.15	0.74*
PLUA (1/mm)	1-Back	FT	17.87 ± 9.34	16.33	0.11	10.14 ± 5.88	10.09	0.1	0.99*
		TD	8.39 ± 2.81	9.03	0.99	5.27 ± 2.42	5.18	0.59	1.19*
		SL	6.98 ± 2.01	7.11	0.93	5.12 ± 2.13	4.68	0.33	0.9*
	3-Back	FT	19.58 ± 10.86	9.91	0.26	11.14 ± 7.56	17.03	0.15	0.9*
		TD	9.70 ± 3.42	10.41	0.61	5.58 ± 2.93	4.95	0.1	1.29*
		SL	7.86 ± 2.49	7.71	0.53	5.33 ± 2.39	4.26	0.29	1.04*

The effect sizes are calculated by Cohen’s d. Values are mean ± SD. The normality tests are calculated by W–S.

*p < 0.05, significantly different compared to skilled athletes.

**p < 0.001, significantly different compared to skilled athletes. ENV, envelope area; WPL, whole path length; MS, mean speed; PLUA, path length per unit area; FT, feet together; TD, tandem; SL, single leg.

For the ENV [*F*(2, 83) = 18.510, *p* < 0.001, η_p_^2^ = 0.287], WPL [*F*(2, 81) = 108.350, *p* < 0.001, η_p_^2^ = 0.720], and MS [*F*(2, 81) = 98.144, *p* < 0.001, η_p_^2^ = 0.681], the main effects of standing posture were significant, indicating that the ENV, WPL, and MS in FT condition was smaller than that in TD and SL conditions (*p*s < 0.01), and the WPL, MS in TD was shorter than that in SL (*p*s < 0.001). Besides, for ENV [*F*(1, 50) = 8.915, *p* = 0.005, η_p_^2^ = 0.162], WPL [*F*(1, 50) = 10.218, *p* = 0.003, η_p_^2^ = 0.182], and MS [*F*(2, 81) = 98.144, *p* < 0.001, η_p_^2^ = 0.681], the main effects of the group were significant, indicating that these indexes in skilled athletes were smaller than non-athletes (*p*s < 0.01). These results suggested better postural control of skilled athletes during dual tasks.

For the PLUA, the main effect of standing posture [*F*(1, 53) = 56.169, *p* < 0.001, η_p_^2^ = 0.550], group [*F*(1, 50) = 23.551, *p* < 0.001, η_p_^2^ = 0.339] and cognitive task [*F*(1, 50) = 4.287, *p* = 0.044, η_p_^2^ = 0.085] were significant, indicating that the PLUA in FT condition (14.68 ± 1.10 1/mm) condition was smaller than that in TD (7.24 ± 0.38 1/mm) and SL (6.32 ± 0.29 1/mm) conditions (*p* < 0.001), and the PLUA in TD was greater than that in SL (*p* = 0.030). Besides, it was greater in skilled athletes (12.16 ± 1.08 1/mm) than non-athletes (7.36 ± 0.89 1/mm) (*p* < 0.001). Moreover, the interaction between the group and cognitive task was marginally significant [*F*(1, 50) = 0.837, *p* = 0.062, η_p_^2^ = 0.282]. Further analyses discovered that the PLUA in 3-back was greater than 1-back (*p* = 0.040) for athletes. The detailed postural control results are available in the Supplementary material.

#### Cognitive control

The ACC, RT, and d′ were included in the statistical analyses (Table 3).

**TABLE 3:** Behavioral data associated with 1-back and 3-back tasks during dual-task.

Measures	Task	Posture	Athletes (*n* = 26)	Median	W-S	Nonathletes (*n* = 26)	Median	W-S	Cohen’s *d*
Accuracy(%)	1-Back	FT	89.44 ± 15.93	92.50	0.21	91.00 ± 4.61	91.67	0.76	–
		TD	94.11 ± 3.45	94.17	0.67	86.43 ± 34.99	92.92	0.58	–
		SL	93.17 ± 4.13	94.17	0.29	92.37 ± 3.53	92.50	0.57	–
	3-Back	FT	84.14 ± 9.72	85.42	0.52	84.62 ± 9.91	85.83	0.28	–
		TD	84.02 ± 8.81	86.25	0.14	85.13 ± 8.51	89.17	0.31	–
		SL	84.03 ± 10.13	87.08	0.61	74.70 ± 34.56	82.92	0.26	–
RT(ms)	1-Back	FT	430.54 ± 58.26	418.89	0.91	420.39 ± 51.17	416.95	0.2	–
		TD	425.45 ± 57.22	417.21	0.23	425.14 ± 64.22	400.97	0.11	–
		SL	431.74 ± 57.40	416.25	0.35	426.12 ± 59.61	412.69	0.18	–
	3-Back	FT	577.53 ± 121.08	566.44	0.61	509.22 ± 97.36	506.76	0.41	–
		TD	570.54 ± 128.62	557.28	0.57	503.12 ± 99.30	506.70	0.34	–
		SL	524.37 ± 104.05	559.56	0.89	428.11 ± 75.70	405.66	0.31	–
d′	1-Back	FT	2.85 ± 0.65	2.73	0.36	2.81 ± 0.60	2.88	0.55	–
		TD	3.09 ± 0.44	3.01	0.13	2.88 ± 0.46	2.93	0.44	0.47*
		SL	3.06 ± 0.50	3.11	0.12	2.61 ± 0.94	2.73	0.28	0.60*
	3-Back	FT	2.17 ± 0.72	2.09	0.91	2.27 ± 0.88	2.05	0.51	–
		TD	2.21 ± 0.60	2.34	0.73	2.10 ± 0.90	2.37	0.13	–
		SL	2.12 ± 0.87	2.14	0.39	1.75 ± 1.02	1.94	0.23	–

The effect sizes are calculated by Cohen’s d. Values are mean ± SD. The normality tests are calculated by W–S.

*p < 0.05, significantly different compared with skilled athletes. ACC, accuracy; RT, reaction time; d′, discriminability.

For the ACC, the main effect of task [*F*(1, 50) = 37.562, *p* < 0.001, η_p_^2^ = 0.429] was significant, indicating that the ACC of 1-back (91.12 ± 1.82%) was higher than 3-back (82.76 ± 2.04%) (*p* < 0.001).

For the RT, the main effect of task [*F*(1, 50) = 111.051, *p* < 0.001, η_p_^2^ = 0.693] and standing posture [*F*(2, 100) = 9.373, *p* < 0.001, η_p_^2^ = 0.158] were significant, indicating that The RT in 3-back (507.99 ± 126.46 ms) was slower than that in 1-back (426.63 ± 57.60 ms) (*p* < 0.001), and the RT in SL (435.08 ± 40.00 ms) condition was shorter than FT (484.02 ± 50.69 ms) (*p* = 0.001) and TD (475.15 ± 71.08 ms) (*p* = 0.004) conditions.

For the d′, the main effect of task [*F*(1, 50) = 87.932, *p* < 0.001, η_p_^2^ = 0.638] and group [*F*(1, 50) = 4.039, *p* = 0.050, η_p_^2^ = 0.075] were significant, indicating that the d′ in 1-back (2.89 ± 0.08) was larger than 3-back (2.12 ± 0.10) (*p* < 0.001), and the d′ in skilled athletes (2.58 ± 0.12) was larger than non-athletes (2.40 ± 0.16) (*p* = 0.050). Besides, the interaction between the group and standing posture was significant [*F*(2, 50) = 2.303, *p* = 0.031, η_p_^2^ = 0.144]. Further analyses revealed that in the TD and SL conditions, the d′ in skilled athletes was larger than non-athletes (*p* = 0.067, *p* = 0.026).

### ERP results

#### N1

**FIGURE 3:**
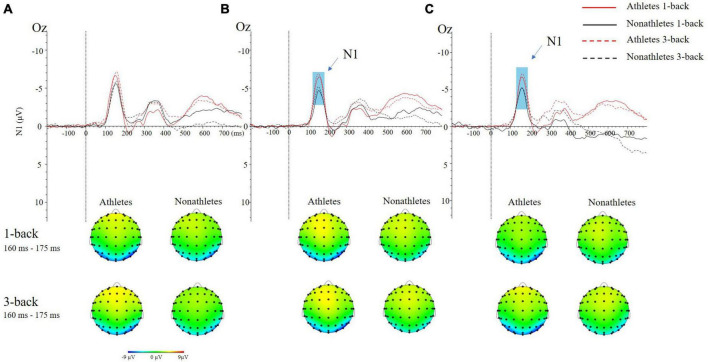
The grand average ERP waveforms (top) and topographical maps (bottom) of two difficulties of N-back tasks in three different standing postures during dual-task at occipital (Oz) site. **(A)** Feet-together standing posture. **(B)** Tandem standing posture. **(C)** Single-leg standing posture.

No main effects and interactions were found in the FT condition (Figure 3A).

In the TD condition, the main effect of group [*F*(1, 50) = 6.496, *p* = 0.014, η_p_^2^ = 0.115] was significant, indicating that the N1 amplitude in skilled athletes (−10.21 ± 0.98 μV) was larger than non-athletes (−7.39 ± 0.98 μV) (*p* = 0.014) (Figure 3B).

In the SL condition, the main effect of group [*F*(1, 50) = 3.069, *p* = 0.066, η_p_^2^ = 0.058] was marginally significant, indicating that the N1 amplitude in skilled athletes (−9.92 ± 1.09 μV) was larger than non-athletes (−7.63 ± 1.27 μV) (*p* = 0.014) (Figure 3C).

#### P2

**FIGURE 4:**
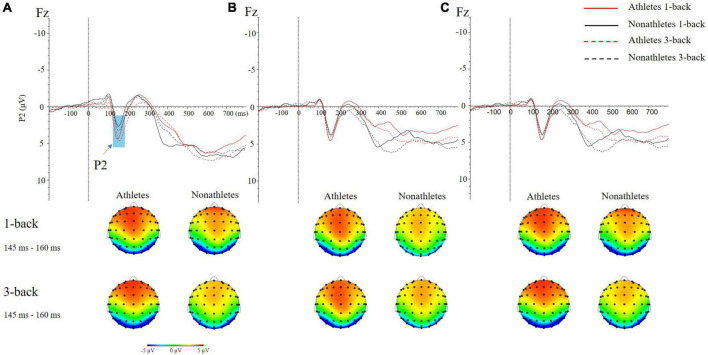
The grand average ERP waveforms (top) and topographical maps (bottom) of two difficulties of N-back tasks in three different standing postures during dual-task at occipital (Fz) site. **(A)** Feet- together standing posture. **(B)** Tandem standing posture. **(C)** Single-leg standing posture.

In the FT condition, the main effect of group [*F*(1, 50) = 6.980, *p* = 0.011, η_p_^2^ = 0.122] was significant, indicating that the P2 amplitude in skilled athletes (4.89 ± 0.55 μV) was larger than non-athletes (3.34 ± 0.65 μV) (*p* = 0.011). The interaction between electrode sites and the group was significant [*F*(3, 50) = 4.261, *p* = 0.006, η_p_^2^ = 0.079]. Besides, the interaction between electrode sites and the task was significant [*F*(3, 64) = 3.180, *p* = 0.026, η_p_^2^ = 0.060]. Further analyses revealed that the P2 amplitude in skilled athletes was larger than non-athletes on Fz, FCz, and Cz electrode sites (*p*s < 0.009). The P2 amplitude in 3-back was smaller than 1-back on Pz (*p* = 0.006) (Figure 4A).

In the TD condition, the interaction between electrode sites and the task was significant [*F*(3, 69) = 4.835, *p* = 0.003, η_p_^2^ = 0.088]. Further analyses revealed that the P2 amplitude in 3-back was smaller than 1-back on Pz (*p* = 0.002) (Figure 4B).

No main effects and interactions were found in the SL condition (Figure 4C).

**FIGURE 5:**
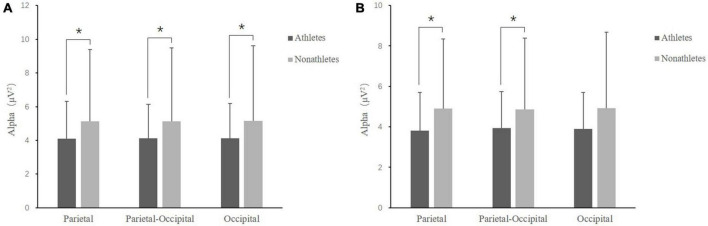
Comparison of alpha band power at various interest areas between skilled athletes and non-athletes for dual-task. **(A)** Feet-together standing posture. **(B)** Tandem standing posture. *p < 0.05 significant difference.

In the FT condition, the main effect of group [*F*(1, 50) = 4.967, *p* = 0.030, η_p_^2^ = 0.090] was significant, indicating that the alpha band power in skilled athletes (4.12 ± 0.41 μV) was smaller than non-athletes (5.14 ± 0.86 μV). The interaction between the group and interest area was significant [*F*(2, 50) = 2.625, *p* = 0.037, η_p_^2^ = 0.250]. Further analyses revealed that the alpha band power in skilled athletes was smaller than non-athletes in three interest areas (*p* < 0.040) (Figure 5A).

In the TD condition, the interaction between the interest area and group was marginally significant [*F*(2, 50) = 2.100, *p* = 0.057, η_p_^2^ = 0.042]. Further analyses revealed that the alpha band power in skilled athletes was smaller than non-athletes in parietal and parieto-occipital interest areas (*p* = 0.068) (*p* = 0.072) (Figure 5B).

In the SL condition, the main effect of task [*F*(1, 50) = 4.519, *p* = 0.0380, η_p_^2^ = 0.083] was significant, indicating that the alpha power in 3-back (3.96 ± 0.29 μV) was smaller than 1-back (4.24 ± 0.33 μV) (*p* = 0.038).

#### EEG results

Moreover, the three-way repeated ANOVA measures 3 (interest areas) × 3 (standing postures) × 2 (cognitive tasks) were performed in each group. Results found that in skilled athletes, the main effect of task [*F*(1, 25) = 5.071, *p* = 0.033, η_p_^2^ = 0.169] was significant, indicating that the alpha power in 3-back (3.856 ± 0.43 μV^2^) was smaller than 1-back (4.35 ± 0.25 μV^2^) (*p* = 0.033).

### Correlation between ERP/EEG and behavioral data

#### N1 amplitude and d′

**FIGURE 6:**
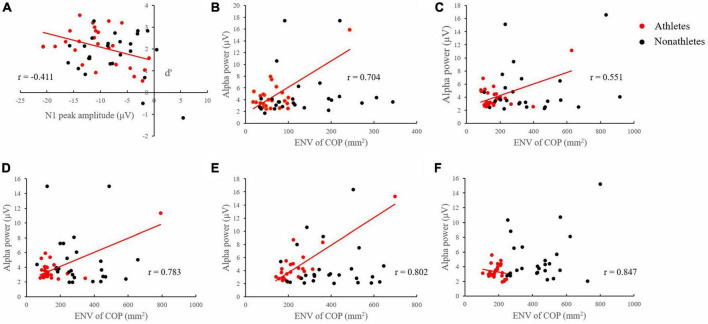
Correlation between EEG and behavioral data. **(A)** Correlation between the N1 component amplitude at Oz electrode site and d′ of skilled athletes and non-athletes in 3-back under SL condition. **(B)** Correlation between the average alpha band power of three interest areas and ENV of skilled athletes and non-athletes in 1-back under FT condition. **(C)** Correlation between the average alpha band power of three interest areas and ENV of skilled athletes and non-athletes in 1-back under TD condition. **(D)** Correlation between the average alpha band power of three interest areas and ENV of skilled athletes and non-athletes in 3-back under TD condition. **(E)** Correlation between the average alpha band power of three interest areas and ENV of skilled athletes and non-athletes in 1-back under SL condition. **(F)** Correlation between the average alpha band power of three interest areas and ENV of skilled athletes and non-athletes in 3-back under SL condition.

In the SL condition, a correlation analysis of skilled athletes revealed a significant negative correlation between N1 amplitude on the Oz electrode site and d′ in 3-back (*p* = 0.037, *r* = −0.411) (Figure 6A).

#### Alpha band power and postural control

In the FT condition, a correlation analysis of skilled athletes revealed a significant positive correlation between the average alpha band power of three interest areas and ENV in 1-back (*p* < 0.001, *r* = 0.704) (Figure 6B).

In the TD condition, a correlation analysis of skilled athletes revealed a significant positive correlation between the average alpha band power of three interest areas and ENV in 1-back (*p* = 0.005, *r* = 0.551) and 3-back (*p* < 0.001, *r* = 0.783) (Figures 6C, D).

In the SL condition, a correlation analysis of skilled athletes revealed a significant positive correlation between the average alpha band power of three interest areas and ENV in 1-back (*p* < 0.001, *r* = 0.802) and 3-back (*p* < 0.001, *r* = 0.847) (Figures 6E, F).

## Discussion

This research compared the postural control of skilled athletes to that of non-athletes. We used the dual-task paradigm to investigate postural control characteristics in skilled athletes under different cognitive processing difficulties. It shed new light on the influence of athletic training on the investment and allocation of attentional resources. Behaviorally, athletes showed higher postural stability when performing dual tasks compared to non-athletes. Their postural control stability increased when performing a more difficult memory task. Besides, the discriminability of skilled athletes was greater than non-athletes when doing dual tasks. Electrophysiologically, athletes showed enhanced N1 and P2 amplitudes and reduced alpha band power during dual tasking compared to non-athletes. Furthermore, there were significant correlations between postural control, cognitive task performance, and EEG signatures in skilled athletes but not non-athletes.

Additionally, this study analyzed 4 COP trajectory indicators: ENV, WPL, MS, and PLUA. Consistent with previous studies on the postural control of skilled athletes (Hain et al., 1999; Bressel et al., 2007; Lamoth et al., 2009), this study discovered that skilled athletes showed greater stability of postural control in various standing postures and working memory tasks than non-athletes under the dual-task situation. The superiority of skilled athletes in this experiment could be related to their balance training in everyday life, which could enhance neuromuscular coordination and joint strength, potentially improving postural control ability (Massion, 1994; Wojtys et al., 2001). In addition, Jaworski et al. (2022, 2023) demonstrated the positive impact of athletic training on balance ability in judo practitioners and badminton athletes, with a particular emphasis on dynamic balance and single-leg stance. The effectiveness of this training may arise from its enhancement of the vestibular organ’s functioning, particularly in proprioceptive sensitivity, which plays a crucial role in stability control within these sports (Fong et al., 2012; Maliński et al., 2017). Therefore, there was a better postural control performance among skilled athletes compared to non-athletes. This advantage is still present in the effect of working memory processing.

Moreover, this study found that the complexity of updating tasks influenced the postural control of skilled athletes. Specifically, the stability of postural control was better in 1-back than in 3-back. Interestingly, this effect was detected only on PLUA, derived from the ratio of WPL to ENV. Generally, the greater the PLUA, the more stable the postural control (Zhang et al., 2011). Zhang et al. (2007) suggested that ENV, WPL, and MS could be used to evaluate the postural control of non-athletes, but their differentiation could be insufficient for athletes. In contrast, PLUA reflected not only the level of postural control but also its regulation. According to the U-shaped hypothesis (Huxhold et al., 2006), this phenomenon could result from attention shifting. Prior studies showed that shifting the focus of overt attention away from postural control could improve it by reducing the interference of a highly automated task (Wulf et al., 1998; Shea and Wulf, 1999; Mcnevin et al., 2003). Technically, skilled athletes need more attentional resources to complete 3-back tasks, which are more difficult than 1-back. The stability of postural control in 3-back is higher for athletes than in 1-back. This result also reflected that the postural control process of skilled athletes was more proficient and automated than non-athletes.

The ERP results in this study also showed differences between athletes and non-athletes. The skilled athletes exhibited greater N1 amplitudes under TD and SL conditions than non-athletes. The N1 component is an early ERP component that reflects the early cortical processing of visual stimuli (Störmer et al., 2009) and is known as an indicator of a discrimination process (Vogel and Luck, 2000). The results suggested that skilled athletes could devote more attentional resources to discriminate the presented stimuli when postural difficulty grew. Regarding the working memory tasks, interesting findings were observed in d′, which reflects reaction sensitivity in cognitive tasks. Skilled athletes demonstrated higher d′ values compared to non-athletes. Furthermore, we found a negative correlation between N1 amplitude and d′ in skilled athletes in SL conditions, meaning that the higher d′ was associated with the larger N1 amplitude in skilled athletes. Therefore, the higher level of discrimination in skilled athletes might be attributable to the larger N1 amplitude in the task. It suggested that athletes deliberately flexibly allocated more attentional resources to enhance discriminability at the early processing stage in the more difficult task, and further implied that the highly practiced postural control in skilled athletes.

Additionally, consistent with prior research, this study discovered that the P2 amplitude decreased as the updating task became more challenging. The P2 component indicates the investment and distribution of attentional resources and is sensitive to task complexity (Maeno et al., 2004; Sugimoto and Katayama, 2013). The difference in P2 amplitude between updating tasks suggested that during dual-tasking, participants could not allocate more attentional resources to the cognitive task as its difficulty increased, which led to the decline in 3-back performance. Meanwhile, in the FT condition, skilled athletes demonstrated a larger P2 component in the frontal lobe (Fz, FCz, and Cz) than non-athletes. This finding suggests that skilled athletes are more inclined to utilize attentional resources in the frontal and parietal areas for cognitive processing, indicating a more focused and economical brain activation during performance (Nakata et al., 2010; Callan and Naito, 2014). However, there was no difference between the two groups in the TD and SL conditions. Potts suggested that frontal P2 is an indicator of stimulus evaluation (Potts, 2004). This result revealed that skilled athletes in the FT condition engaged more attentional resources in the stimulus evaluation process than non-athletes. Interestingly, this pattern contradicted the N1 component, which showed differences between groups in the TD and SL conditions. This phenomenon could be a result of the responding strategy of athletes. In the simple postural condition, skilled athletes could intentionally allocate attentional resources to updating tasks for stimulus evaluation. However, as the difficulty of the balance task increased, they tended to devote their limited attentional resources to early stimuli to perform well in dual-task. In summary, the results of the P2 component further reflected the flexibility of attentional resource allocation in skilled athletes.

Moreover, this study also examined the EEG spectral power in the alpha band. The results demonstrated that the alpha power of skilled athletes was lower than non-athletes in FT and TD. Neuper and Pfurtscheller (2001) suggested that the alpha band reflects energetic processes such as arousal and attentional level. It has been linked to inhibitory processes. The attenuated alpha power indicates efficient cognitive-motor processes during task performance (Cheng et al., 2015; Ghasemian et al., 2017). Thus, our results indicated that athletes had a lower activation level in the brain than non-athletes under the two postural conditions. This phenomenon could be due to the neural efficiency of athletes. Long-term systematic exercise training could lead to plasticity changes in the structure and function of the human brain, enhancing neural efficiency (Clare and Hugh, 2005). It mainly manifests in reducing the demand for external attention and the dependence on executive function. Percio et al. (2010) demonstrated that karate athletes had lower levels of low alpha and high alpha in the primary motor and lateral and medial premotor areas than non-athletes. They also revealed that the low alpha and high alpha event-related desynchronization (ERD) were lower in skilled pistol shooters than in non-athletes over the whole scalp during shooting practice (Percio et al., 2009). These studies demonstrated the decreased cortical activity of skilled athletes during motor performance. Our correlation result confirmed this claim. A positive correlation existed between alpha power and ENV in skilled athletes, i.e., the more stable they stand, the lower brain activity. Besides, the alpha power of skilled athletes was lower in 3-back than in the 1-back condition, which is consistent with our behavioral result that athletes had a higher postural control level in 3-back compared with 1-back. These results revealed a high level of automation in postural control of skilled athletes with lower cortical activation than non-athletes. This mechanism helps optimize the allocation of attentional resources and reduces interference in dual-tasking.

In conjunction with the findings above, skilled athletes demonstrated superior performance in dual-task conditions and exhibited distinctive allocation and utilization of attentional resources. This outcome may be attributed to their regular skill training, which involves the simultaneous recall and extraction of action information while maintaining stable postures. This parallel resembles the demands of dual-task training. Previous studies have indicated that working memory training can impact the top-down modulation of attentional processes and promote cognitive plasticity (Salminen et al., 2016; Jaquerod et al., 2020). Furthermore, N-back training has been shown to enhance functional connectivity of the right inferior frontal gyrus, leading to improved working memory efficiency (Salminen et al., 2020). The training regimens of skilled athletes may exert effects on neural plasticity, thereby conferring advantages in attentional processing.

In summary, this study demonstrated that athletes of skill-oriented sports had greater postural control ability than non-athletes when performing dual tasks. However, it had some limitations that should be considered. First, only dual tasks were performed in the experiments. Although we found the differences between the two groups on postural control and N-back tasks, the participants’ performance in single tasks remained unknown. Moreover, this study did not cover the relationship between long-term athletic training in reactive sports and neural efficiency in the brain, which has been widely discussed in the literature. Therefore, future research should also explore reactive sports in order to elucidate the specificity of skill-oriented sports training on postural control processes. Furthermore, to further our comprehension of the mechanism behind the allocation of attentional resources in skill-oriented sports, future studies should explore cause-and-effect relationships between skilled athletes’ sports level and postural control ability under multi-task conditions. This would offer valuable insights for optimizing training programs in skill-oriented sports.

## Conclusion

In conclusion, skilled athletes were more effective at postural control due to their highly automated control abilities. This advantage may imply a more rational allocation of attentional resources in skilled athletes, such as decreasing postural control consumption and obtaining more attentional resources in stimulus discrimination and evaluation in cognitive tasks, allowing them to attain superior and steadier cognitive and balance performance than non-athletes. Optimal postural control training in skill-oriented sports should foster balance control and promote the “neural economy” for attentional resource recruitment by appropriate multitask intervention.

## Data availability statement

The original contributions presented in this study are included in the article/Supplementary material, further inquiries can be directed to the corresponding author.

## Ethics statement

The studies involving humans were approved by the Ethics Committee of the Shanghai University of Sports. The studies were conducted in accordance with the local legislation and institutional requirements. The participants provided their written informed consent to participate in this study. Written informed consent was obtained from the individual(s) for the publication of any potentially identifiable images or data included in this article.

## Author contributions

JC designed experiments, collected the data, analyzed the data, and wrote this manuscript. AK revised the manuscript. YL analyzed the data and discussed the results. All authors contributed to the article and approved the submitted version.
