# Supplementary material for: Effective utilization of attentional resources in postural control in athletes of skill-oriented sports: an event-related potential study
Source: Front Hum Neurosci. 2023 Aug 24;17:1219022. doi: 10.3389/fnhum.2023.1219022 (PMC10483146; doi:10.3389/fnhum.2023.1219022)
Supplement: Supplementary file 1 [file Data_Sheet_1.docx]

Supplementary Material

Effective utilization of attentional resources in postural control in athletes of skill-oriented sports: An event-related potential study

Jiacheng Chen^a^, Alex Pak Ki Kwok^b^, Yanan Li^c,^*

*^a^College of Education for the Future, Beijing Normal University at Zhuhai 519087, Zhuhai, China*

*^b^Data Science and Policy Studies Programme, Faculty of Social Science, The Chinese University of Hong Kong, 999077, Hong Kong, China*

*^c^Physical Education Department, Zhuhai Campus of Jinan University 519070, Zhuhai, China*

*** Correspondence:**

Yanan Li

liyanan@jnu.edu.cn

# Supplementary Data

**Behavioral results of Postural control**

The COP trajectory: ENV, WPL, MS, and PLUA were included in statistical analyses (Table 1).

For the ENV, the main effect of standing posture (*F* (2,83) =18.510, *p* < 0.001, *η*_p_^2^ = 0.287) and group (*F* (1,50) = 8.915, *p* = 0.005, *η*_p_^2^ = 0.162) were significant, indicating that the ENV in FT (100.29 ± 10.75mm^2^) condition was smaller than that in TD (304.76 ± 47.24 mm^2^) and SL (358.91 ± 41.22 mm^2^) conditions (*p* < 0.001). The ENV in skilled athletes (174.05 ± 36.74 mm^2^) was smaller than that in nonathletes (324.42 ± 51.83 mm^2^) (*p* = 0.005). However, there was no significant interaction between groups, standing postures, and cognitive tasks.

For the WPL, the main effect of standing posture (*F* (2,81) =108.350, *p* < 0.001, *η*_p_^2^ = 0.720) and group (*F* (1,50) = 10.218, *p* = 0.003, *η*_p_^2^ = 0.182) were significant, indicating that the WPL in FT (814.41 ± 40.74 mm) condition was smaller than that in TD (1315.59 ± 45.57 mm) and SL (1637.61 ± 56.36 mm) conditions (*p* < 0.001). Besides, the WPL in TD was shorter than that in SL (*p* < 0.001). The WPL in skilled athletes (1145.34 ± 39.21 mm) was smaller than that in nonathletes (1365.10 ± 88.27 mm) (*p* = 0.003). However, there was no significant interaction between group, standing posture, and cognitive tasks.

For the MS, the main effect of standing posture (*F* (2,81) =98.144, *p* < 0.001, *η*_p_^2^ = 0.681) and group (*F* (1,50) = 9.709, *p* = 0.003, *η*_p_^2^ = 0.174) were significant, indicating that the MS in FT (13.71 ± 0.73 mm/s) condition was smaller than that in TD (22.12 ± 0.80 mm/s) and SL (27.54 ± 0.99 mm/s) conditions (*p* < 0.001). Besides, the MS in TD was shorter than that in SL (*p* < 0.001). The MS in skilled athletes (19.26 ± 0.67 mm/s) was smaller than that in nonathletes (23.00 ± 1.55 mm/s) (*p* = 0.003). However, there was no significant interaction between groups, standing postures, and cognitive tasks.

For the PLUA, the main effect of standing posture (*F* (1,53) =56.169, *p* < 0.001, *η*_p_^2^ = 0.550), group (*F* (1,50) = 23.551, *p* < 0.001, *η*_p_^2^ = 0.339) and cognitive task (*F* (1,50) = 4.287, *p* = 0.044, *η*_p_^2^ = 0.085) were significant, indicating that the PLUA in FT (14.68 ± 1.10 1/mm) condition was smaller than that in TD (7.24 ± 0.38 1/mm) and SL (6.32 ± 0.29 1/mm) conditions (*p* < 0.001). Besides, the PLUA in TD was shorter than that in SL (*p* = 0.030). The PLUA in skilled athletes (12.16 ± 1.08 1/mm) was smaller than that in nonathletes (7.36 ± 0.89 1/mm) (*p* < 0.001), and the PLUA in 3-back (9.87 ± 0.58 1/mm) was greater than that in 1-back (8.96 ± 0.46 1/mm) (*p* = 0.044). The interaction between standing posture and group was significant (*F* (1,50) = 6.305, *p* = 0.012, *η*_p_^2^ = 0.121). Further analyses revealed that in athletes, the PLUA in TD was greater than in SL (*p* = 0.005). The interaction between group and cognitive task was marginally significant (*F* (1,50) = 0.837, *p* = 0.062, *η*_p_^2^ = 0.282). Further analyses also discovered that the PLUA in 3-back was greater than that in 1-back (*p* = 0.040) for athletes.

**
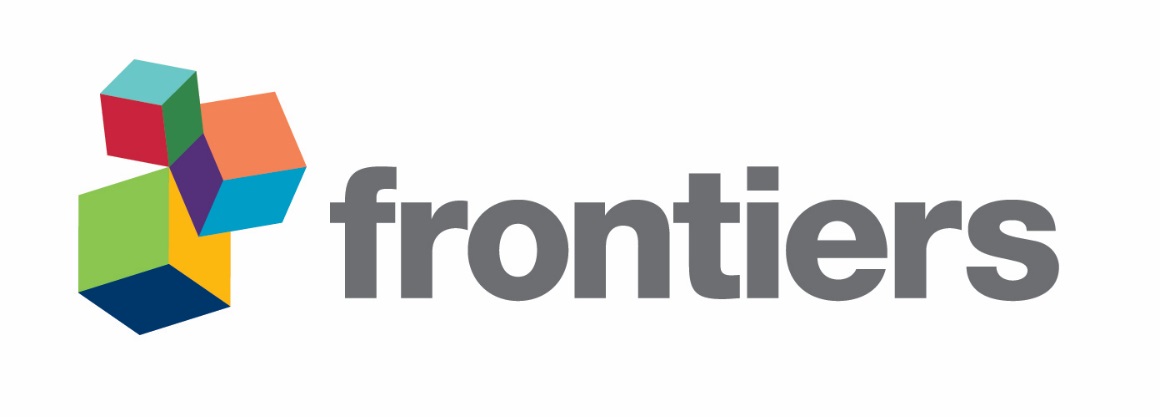
**
